# Food Variety and Unhealthy Food Consumption Among International Students in Hungary: Associations with Sociodemographic and Behavioural Factors

**DOI:** 10.3390/nu18142277

**Published:** 2026-07-11

**Authors:** Zibuyile Mposula, Tünde Pacza, Judit Szepesi, Endre Máthé

**Affiliations:** 1 Doctoral School of Food Sciences, Faculty of Agricultural and Food Sciences and Environmental Management, University of Debrecen, Böszörményi Str. 128, 4032 Debrecen, Hungary; 2 Institute of Nutrition Science, Faculty of Agricultural and Food Sciences and Environmental Management, University of Debrecen, Böszörményi Str. 128, 4032 Debrecen, Hungary; pacza.tunde@unideb.hu (T.P.); szepesi@agr.unideb.hu (J.S.); 3 Department of Life Sciences, Faculty of Medicine, Vasile Goldis Western University of Arad, L. Rebreanu Str. 86, 310414 Arad, Romania

**Keywords:** food variety score, unhealthy food consumption, diet quality, food frequency questionnaire, international students, Hungary

## Abstract

**Background:** International students are exposed to new food environments that may influence their dietary behaviours. While dietary diversity has been widely studied, less attention has been given to food variety, which reflects the number of individual food items consumed. This study assessed food variety, its relationship with unhealthy food consumption, and associated sociodemographic and behavioural factors among international students in Hungary. **Methods**: A cross-sectional survey was conducted among 380 international students enrolled in Hungarian higher education institutions. Data were collected using a structured questionnaire incorporating a Food Frequency Questionnaire (FFQ). FVS and UFCS were calculated, and associations were examined using descriptive statistics, Pearson correlation, and multivariable linear regression analyses. **Results**: The mean FVS was 62.7 ± 26.7. Cereals, vegetables, and milk and dairy products were consumed by all participants, whereas fish and other seafood (61.6%) and white tubers and roots (67.9%) were the least frequently consumed food groups. A very strong positive correlation was observed between FVS and UFCS (r = 0.930, *p* < 0.001). This association remained strong after excluding unhealthy food items from the FVS (aFVS; r = 0.862, *p* < 0.001). In multivariable analyses, higher monthly income and employment were positively associated with FVS and UFCS, whereas scholarship status, postgraduate study, and following a special diet were negatively associated with both outcomes. **Conclusions**: Food variety among international students in Hungary varied considerably and was strongly associated with unhealthy food consumption, indicating that greater food variety did not necessarily correspond to healthier dietary patterns. These findings highlight the importance of considering both the diversity and nutritional quality of foods consumed when assessing dietary behaviours and designing nutrition interventions for international students.

## 1. Introduction

International students studying abroad are exposed to substantial dietary and lifestyle changes that may influence their eating behaviours and overall nutritional status [1,2]. As globalization continues to expand access to international higher education, increasing numbers of students are pursuing academic opportunities outside their home countries, resulting in diverse and multicultural student populations across universities worldwide [3]. International students often face challenges in adapting to new food environments, which can affect their health, well-being, and academic performance [4]. Relocation to a foreign country may alter food accessibility, affordability, and cultural dietary practices, thereby influencing eating behaviours and overall dietary patterns [5]. These dietary transitions may have important implications for health, well-being, and academic performance among international students.

Dietary diversity (DD), commonly defined as the consumption of a variety of food groups over a given reference period [6], is widely recognised as an indicator of dietary quality and nutrient adequacy [7]. Nevertheless, while DD reflects the inclusion of different food groups, it may not fully capture the variety of individual food items consumed within those groups. Food variety, which reflects the number of different individual foods consumed, may provide a more detailed understanding of dietary behaviour and eating patterns [8]. Importantly, greater food variety does not necessarily indicate healthier dietary intake, particularly in food environments where highly processed and energy-dense foods are widely accessible [9,10].

Although dietary diversity and food variety are related concepts, they capture different dimensions of dietary intake. Dietary diversity focuses on the consumption of foods across broad food groups and is commonly used as a proxy for nutrient adequacy. In contrast, FVS quantifies the number of individual food items consumed within and across food groups, thereby providing a more detailed measure of dietary exposure. Consequently, individuals with similar levels of dietary diversity may differ substantially in the variety of individual food items consumed. In populations undergoing dietary transition, such as international students adapting to new food environments, FVS may offer complementary insight into food choice patterns and dietary behaviours that may not be fully captured by food-group-based indicators alone.

Socio-demographic factors such as age, income, education, employment, and access to resources have consistently been associated with dietary quality and food choices [11,12]. Higher socioeconomic status has been linked to more varied and nutritionally adequate diets characterised by greater consumption of fruits, vegetables, and high-quality protein sources [13,14], whereas lower-income groups often experience financial barriers that limit access to nutritious foods and increase reliance on inexpensive, energy-dense, and processed products [15]. Among international students, these influences may be compounded by financial constraints, unfamiliar food environments, cultural dietary adaptation, and limited access to preferred foods from their home countries, all of which may affect both food variety and dietary quality [16]. Despite growing research on student nutrition, relatively few studies have examined the factors associated with food variety among international students, particularly within the context of dietary transition and unhealthy food consumption [17].

In addition to food variety, unhealthy food consumption has become an important dietary concern among university students. Diets characterised by frequent consumption of processed meats, sugary snacks, fried foods, sugar-sweetened beverages, and other highly processed foods have been associated with poor dietary quality and adverse health outcomes. Previous studies have shown that university students frequently rely on convenient and affordable food options due to academic pressures, financial limitations, and time constraints, which may contribute to increased consumption of unhealthy foods [18]. Within transitional food environments, increased food variety may therefore coexist with greater consumption of unhealthy foods, creating a paradox in which broader food exposure does not necessarily reflect healthier dietary patterns [19].

Hungary presents a unique context for examining dietary behaviours among international students due to its distinct culinary environment [20]. Traditional Hungarian cuisine includes a range of ingredients and preparation methods that may differ substantially from those of students’ home countries, potentially influencing dietary adaptation [21]. Additionally, the availability and affordability of familiar foods may vary, requiring students to modify their eating habits [22]. These dietary adaptations may influence not only the variety of foods consumed, but also the quality of dietary choices made within the host food environment [23].

Investigating the relationship between sociodemographic characteristics, food variety, and unhealthy food consumption among international students in Hungary is important for understanding potential nutritional inequalities within this population. Therefore, this study aimed to examine the associations of sociodemographic and behavioural characteristics with FVS and unhealthy food consumption among international students in Hungary by comparing food variety across participant characteristics, evaluating the relationship between FVS and UFCS, and identifying factors associated with these dietary outcomes.

## 2. Materials and Methods

### 2.1. Study Design and Population

The study used a cross-sectional survey design to assess food variety and unhealthy food consumption among international students in Hungary and to examine their associations with sociodemographic and behavioural characteristics. The study sample comprised international students enrolled at Hungarian higher education institutions. Participants were recruited using a combination of purposive and convenience sampling approaches and voluntarily participated in the study after meeting the eligibility criteria and providing informed consent. As a non-probability sampling method was used, the findings may not be fully representative of all international students in Hungary.

### 2.2. Sample Calculation

The required sample size was estimated using Cochran’s formula for proportions [24], assuming a 95% confidence level, a 5% margin of error, and a proportion of 0.5 to maximise variability. The initial sample size was calculated as 384 participants. Given the finite population of international students in Hungary, a finite population correction was applied, resulting in a final minimum sample size of 381 participants. Although the calculated minimum sample size was 381 participants, 380 complete questionnaires were available for the final analysis after excluding one incomplete questionnaire. This difference was considered negligible and unlikely to affect the statistical precision of the study.

### 2.3. Inclusion Criteria

Eligible participants included male and female international students aged 18 to 40 years, enrolled in Hungarian higher education institutions. Participants were required to have a sufficient understanding of the English language, provide informed consent, and agree to complete the questionnaire. Students with specific dietary practices or restrictions, including vegetarian or vegan diets, food allergies, or religious dietary practices, were not excluded, as these reflect real-world dietary behaviours within the study population.

Exclusion criteria included individuals who were not enrolled as international students at a Hungarian higher education institution, were younger than 18 years or older than 40 years, were unable to complete the questionnaire in English, declined to provide informed consent, or submitted incomplete questionnaires with substantial missing data.

### 2.4. Data Collection Tools

The survey was administered online using Google Forms^®^ and consisted of two main components: (a) a sociodemographic questionnaire and (b) a Food Frequency Questionnaire (FFQ). The sociodemographic questionnaire collected information on participants’ demographic, socioeconomic, and behavioural characteristics, while the FFQ was designed to assess the consumption of individual food items over the previous seven days. Participants were asked to indicate whether they had consumed each food item during the reference period using dichotomous response options (yes/no). The questionnaire included 101 individual food items selected to represent foods commonly available within the Hungarian food environment. The food items also captured the culturally diverse dietary practices of international students.

### 2.5. Development and Validation of the Questionnaire

The questionnaire consisted of a sociodemographic section and a dietary assessment section based on an FFQ adapted from previously validated tools [25]. The FFQ incorporated 12 food groups recommended by the Food and Nutritional Technical Assistance III Project (FANTA) [26] and included 101 individual food items used to assess food consumption patterns among participants. The food items were selected through adaptation of previously validated FFQs. They were chosen to reflect both commonly consumed foods within Hungary and foods frequently consumed by culturally diverse international student populations. The final list included staple foods, animal-source foods, fruits, vegetables, dairy products, beverages, snack foods, and convenience foods across the 12 FANTA food groups. The same FFQ was used to derive both the FVS and the UFCS. The FFQ was administered using a seven-day reference period. The FVS was calculated using all 101 food items included in the FFQ, whereas the UFCS was calculated using a predefined subset of food items classified as unhealthy.

The questionnaire was adapted to reflect both the Hungarian food environment and the culturally diverse dietary practices of international students. To ensure clarity, comprehensiveness, and contextual relevance, the instrument was pretested among a small sample of international students (*n* = 10) who met the study eligibility criteria. Minor modifications were made based on participant feedback to improve question clarity and interpretation.

In addition, the questionnaire was reviewed by an independent expert panel comprising two senior academics to assess content and face validity. As the FFQ was adapted from previously validated instruments, additional psychometric validation, including internal consistency and test–retest reliability, was not conducted within this study. However, the use of validated source instruments, expert review, and pilot testing supported the suitability of the questionnaire for the study objectives.

The final version of the questionnaire was administered online using Google Forms^®^ and consisted of two sections: the sociodemographic questionnaire and the FFQ.

### 2.6. Data Collection

Recruitment and data collection were conducted by the researcher between June 2022 and January 2024. Prior to data collection, the necessary permissions and approvals were obtained from the relevant authorities at participating institutions.

A combination of purposive and convenience sampling approaches was used to recruit participants. International students who met the eligibility criteria were identified through collaboration with university staff, lecturers, student representatives, and institutional communication channels. Information about the study and scheduled data collection sessions was disseminated through official university channels.

Data collection took place at designated locations within participating institutions, including lecture venues, university facilities, and other agreed meeting points accessible to international students. Eligible students were invited to participate voluntarily and were asked to complete the questionnaire within a designated period. By submitting the completed questionnaire, participants provided informed consent to participate in the study.

### 2.7. Statistical Analysis

Data were analysed using Microsoft Excel^®^ (version 16.89.1) for preliminary data management and Statistical Package for the Social Sciences^®^ (SPSS) software (version 29.0.0.0) for statistical analyses. Descriptive statistics were used to summarise participant characteristics and are presented as frequencies, percentages, and means ± standard deviations. Differences in mean FVS across sociodemographic and behavioural variables were assessed using independent samples *t*-tests and one-way analysis of variance (ANOVA), as appropriate. Pearson correlation analysis was used to examine the relationships between FVS, aFVS, and UFCS. Multiple linear regression analyses were conducted to examine factors associated with FVS, aFVS, and UFCS. Statistical significance was set at *p* < 0.05. Variables that were not significantly associated with FVS in bivariate analysis were not included in the multivariable regression model. This was done to improve model parsimony and reduce unnecessary model complexity. Assumptions of normality for the regression models were assessed using graphical methods, including histograms and normal P–P plots (Supplementary Figures S2–S4). Multicollinearity was assessed using variance inflation factors (VIFs).

### 2.8. Ethical Consideration

This study adhered to the principles outlined in the Declaration of Helsinki, with all procedures involving human subjects receiving approval from the Institute of Nutrition and Food Science Research Ethics Committee. Written informed consent was obtained from all participants prior to participation, and all institutional and national guidelines were strictly observed. Participants were informed of their right to withdraw from the study at any time without consequence. To ensure confidentiality, all data were anonymised, and participants were assigned unique identification numbers.

### 2.9. Definitions

FVS: A quantitative measure representing the total number of unique food items consumed by an individual over a specified reference period [8]. Each distinct food item is counted once, and the score reflects overall dietary variety. Unlike DD indicators that assess consumption across food groups, FVS captures variation in the number of individual food items consumed both within and across food groups, providing a more detailed assessment of dietary exposure and food choice patterns. In this study, the FVS ranged from 0 to 101 and was derived based on food items reported across 12 food groups, consistent with established approaches for assessing dietary variety.

Unhealthy food consumption score (UFCS): A composite dietary indicator derived from 30 food items identified as discretionary foods, processed foods, sugar-sweetened beverages, alcoholic beverages, and fast foods. The selection of these food items was informed by the World Food Programme Food Consumption Score (FCS) framework [27]. Similarly to the FCS methodology, the UFCS was constructed by assigning one point for the consumption of each predefined unhealthy food item reported in the FFQ. The score reflected the consumption of processed and nutritionally less favourable food items. These included high-fat, high-sugar, and high-salt foods classified according to the NOVA food classification system for ultra-processed foods [28]. Individual food items were coded as Yes = 1 and No = 0. Item scores were summed to obtain a total UFCS ranging from 0 to 30, with higher scores indicating greater consumption of unhealthy food items.

## 3. Results

### 3.1. Socio-Demographic Characteristics of the Participants

As shown in Table 1, a total of 380 international students participated in the study. The sample was almost evenly distributed by sex, comprising 51% males and 49% females. More than half of the participants were aged 18–25 years (54%), followed by those aged 26–30 years (34%), while 12% were older than 30 years. The majority of participants originated from Africa (64%), followed by Asia (27%), Europe (7%), and the Americas (2%).

Most participants lived in shared paid accommodation (45%), followed by renting alone (26%) and university dormitories (24%), whereas only 5% lived in shared accommodation without payment. Slightly more than half of the participants were scholarship recipients (55%), while 45% were not receiving scholarships. Undergraduate students accounted for 53% of the sample, with the remaining 47% enrolled in postgraduate programmes.

A substantial proportion of participants were unemployed (71%), whereas 29% were employed. Monthly income varied, with more than half of the participants (54%) reporting an income of 101,000–200,000 HUF, followed by ≤100,000 HUF (20%), 201,000–300,000 HUF (19%), and >300,000 HUF (7%). The distribution of FVS and UFCS among participants is presented in the following sections.

### 3.2. Food Variety Score Distribution

**Figure 1:**
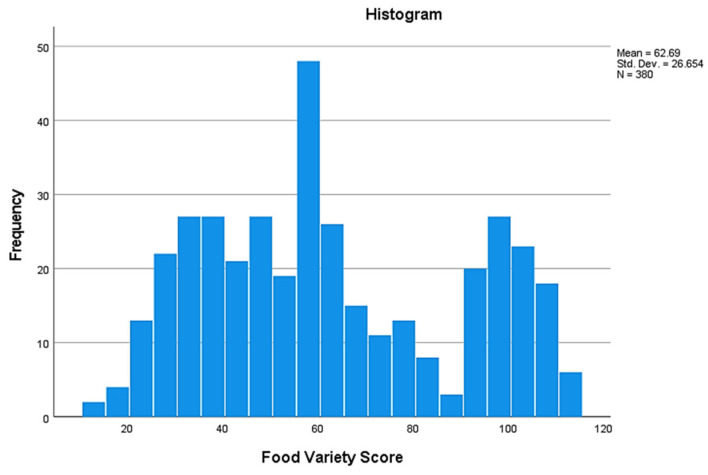
Distribution of food variety score among international students in Hungary (*n* = 380).

As shown in Figure 1, the mean FVS among participants was 62.69 ± 26.65, with values ranging from 13 to 111. The median FVS was 58.00. Additional descriptive statistics for the FVS, aFVS, and UFCS are presented in Supplementary Table S2. Although the Shapiro–Wilk test indicated statistically significant departures from normality for all three scores (*p* < 0.001), visual inspection of the FVS boxplot (Supplementary Figure S1), Normal Q–Q plots (Supplementary Figures S2–S4), and regression diagnostic plots (Supplementary Figures S5–S10) indicated only minor departures from normality. Given the large sample size, parametric analyses were considered appropriate.

### 3.3. Food Consumption Habits Based on Food Groups

**Figure 2:**
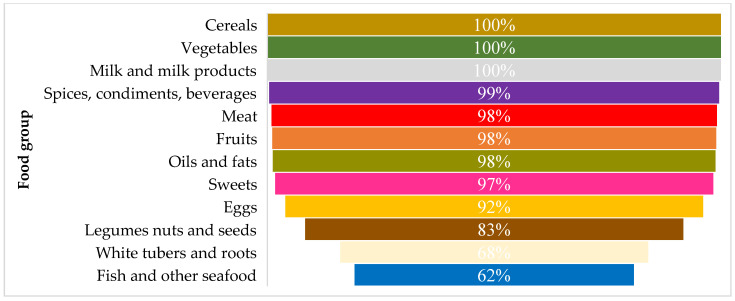
Consumption of foods by participants based on food groups.

Figure 2 represents food group consumption habits based on the 12 food groups used in the FANTA III Project household dietary diversity score (HDDS) framework [26]. The most commonly consumed food groups were cereals, vegetables, and milk and dairy products, all reported by 100% of participants. Lower consumption rates were observed for fish and other seafood (61.6%) and white tubers and roots (67.9%).

### 3.4. Most Consumed Food Items by Participants

**Figure 3:**
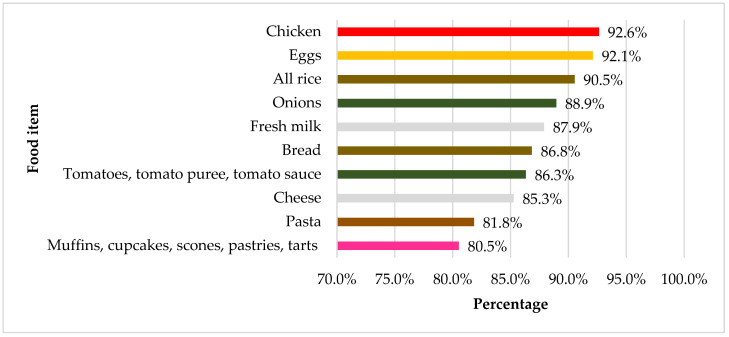
Most consumed food items by participants.

As shown in Figure 3, chicken was the most frequently consumed food item, reported by 92.6% of participants, followed by eggs (92.1%) and rice (90.5%). Other commonly consumed items included onions (88.9%), fresh milk (87.9%), bread (86.8%), tomatoes and tomato-based products (86.3%), cheese (85.3%), pasta (81.8%), and baked goods such as muffins, cupcakes, scones, pastries, and tarts (80.5%).

### 3.5. Least Consumed Food Items by Participants

**Figure 4:**
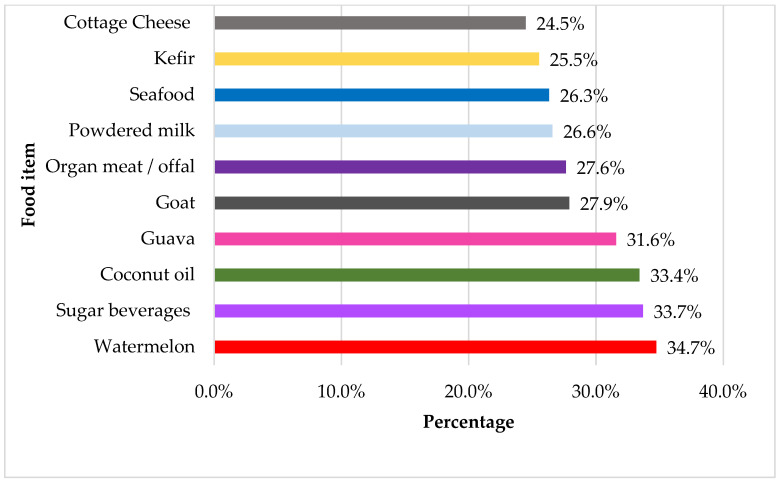
Least consumed food items by participants.

Figure 4 indicates that cottage cheese was the least consumed item, reported by 24.5% of participants. Other minimally consumed items included kefir (25.5%), seafood (26.3%), powdered milk (26.6%), organ meat or offal (27.6%), and goat meat (27.9%). Lower consumption was also observed for guava (31.6%), coconut oil (33.4%), sugar-sweetened beverages (33.7%), and watermelon (34.7%).

### 3.6. Food Variety Score Across Sociodemographic and Behavioural Characteristics

As shown in Table 2, mean FVS differed significantly across monthly income categories (*p* < 0.001), with the lowest mean observed among participants earning ≤100,000 HUF (42.5 ± 17.1). Participants earning 101,000–200,000 HUF had the highest mean FVS (70.3 ± 27.7), followed by those earning >300,000 HUF (65.2 ± 19.3) and 201,000–300,000 HUF (61.7 ± 23.0).

Employed participants had significantly higher FVS (70.3 ± 27.8) than unemployed participants (59.5 ± 25.6, *p* < 0.001). Similarly, participants who were not on scholarship had higher FVS (69.7 ± 28.0) than scholarship recipients (57.0 ± 24.1, *p* < 0.001). Undergraduate students also had significantly higher FVS (66.7 ± 27.7) than postgraduate students (58.3 ± 24.7, *p* = 0.002).

Participants who had received nutrition education reported higher FVS (68.1 ± 27.9) than those without nutrition education (61.0 ± 26.1, *p* = 0.035). In contrast, participants following a special diet had significantly lower FVS (50.0 ± 21.1) than those not following a special diet (68.3 ± 27.0, *p* < 0.001).

### 3.7. The Association Between Food Variety Score and Unhealthy Food Consumption Score

As shown in Table 3, a very strong positive correlation was observed between FVS and UFCS (r = 0.930, *p* < 0.001). After excluding the 30 food items included in the UFCS from the FVS, a strong positive correlation remained between aFVS and UFCS (r = 0.862, *p* < 0.001).

### 3.8. Factors Associated with Food Variety Score

Table 4 presents the multivariable linear regression analysis of factors associated with the FVS. The overall model was statistically significant (F(6, 373) = 17.81, *p* < 0.001), explaining 22.3% of the variance in FVS (R^2^ = 0.223; adjusted R^2^ = 0.210). Higher monthly income (β = 3.82, 95% CI: 0.69–6.94, *p* = 0.017) and being employed (β = 11.80, 95% CI: 6.19–17.41, *p* < 0.001) were positively associated with FVS. In contrast, scholarship recipients (β = −10.63, 95% CI: −15.57 to −5.69, *p* < 0.001), postgraduate students (β = −7.99, 95% CI: −13.02 to −2.95, *p* = 0.002), and participants following a special diet (β = −14.92, 95% CI: −20.25 to −9.60, *p* < 0.001) had significantly lower FVS. Although nutrition education was positively associated with FVS, the association did not reach statistical significance after adjustment (β = 5.70, 95% CI: −0.07 to 11.47, *p* = 0.053).

To assess the robustness of these findings, a sensitivity analysis was conducted using the adjusted aFVS, which excluded the food items included in the Unhealthy Food Consumption Score. The overall model remained statistically significant (F(6, 373) = 16.42, *p* < 0.001), explaining 20.9% of the variance in aFVS (R^2^ = 0.209; adjusted R^2^ = 0.196). The direction and magnitude of the associations were largely consistent with those observed for the FVS. Higher monthly income (β = 2.78, 95% CI: 0.50–5.07, *p* = 0.017) and being employed (β = 8.77, 95% CI: 4.66–12.88, *p* < 0.001) remained positively associated with aFVS, whereas scholarship recipients (β = −7.50, 95% CI: −11.11 to −3.88, *p* < 0.001), postgraduate students (β = −5.38, 95% CI: −9.06 to −1.69, *p* = 0.004), and participants following a special diet (β = −10.10, 95% CI: −14.00 to −6.21, *p* < 0.001) continued to have significantly lower scores. Unlike the primary analysis, nutrition education was independently associated with higher aFVS after adjustment (β = 4.24, 95% CI: 0.02–8.46, *p* = 0.049).

### 3.9. Factors Associated with the Unhealthy Food Consumption Score

Table 5 presents the multivariable linear regression analysis of factors associated with the UFCS. The overall model was statistically significant (F(6, 373) = 17.04, *p* < 0.001), explaining 21.5% of the variance in UFCS (R^2^ = 0.215; adjusted R^2^ = 0.203). Higher monthly income (β = 1.04, 95% CI: 0.08–1.99, *p* = 0.034) and being employed (β = 3.03, 95% CI: 1.31–4.75, *p* < 0.001) were positively associated with UFCS. In contrast, scholarship recipients (β = −3.14, 95% CI: −4.65 to −1.63, *p* < 0.001), postgraduate students (β = −2.61, 95% CI: −4.15 to −1.07, *p* < 0.001), and participants following a special diet (β = −4.82, 95% CI: −6.45 to −3.19, *p* < 0.001) had significantly lower UFCS. Although nutrition education was positively associated with UFCS, the association did not remain statistically significant after adjustment (β = 1.46, 95% CI: −0.30 to 3.23, *p* = 0.104).

## 4. Discussion

### 4.1. Food Variety Score Distribution

The mean FVS observed among participants indicates substantial variability in the number of individual food items consumed within the study population. The relatively wide distribution of FVS values may reflect heterogeneity in dietary exposure, food accessibility, cultural dietary adaptation, and individual food preferences among international students living in a foreign food environment. Visual inspection of the FVS distribution also suggested considerable variation in food variety among participants, further reflecting the DD experiences within this multicultural student population. These findings suggest that international students may experience varying levels of dietary exploration and adaptation within the host-country food environment [29].

Direct comparison of the FVS observed in the present study with values reported elsewhere is challenging. FVS estimates are highly dependent on the dietary assessment methodology used. Important considerations include the number of food items included in the questionnaire, the reference period assessed, and the characteristics of the study population. Nevertheless, previous research has recognised FVS as a useful indicator of dietary exposure and dietary adequacy when used alongside complementary dietary assessment measures [8]. The relatively high mean FVS observed in the present study may reflect the diverse food environment encountered by international students in Hungary and their exposure to both culturally familiar and host-country food choices.

Visual inspection of the FVS distribution suggested a possible bimodal pattern, indicating that international students may comprise distinct dietary subgroups with different levels of food variety. Although exploring these underlying dietary patterns was beyond the scope of the present study, future research could apply clustering approaches, such as cluster analysis or latent class analysis, to identify dietary profiles and better understand heterogeneity in food choice behaviours among international students.

### 4.2. Food Consumption Patterns

The findings of this study indicate that cereals, milk and dairy products, and vegetables were consumed by all participants (100%), suggesting that these food groups form the foundation of dietary intake among international students in Hungary. The universal consumption of these food groups may reflect their widespread availability and affordability. It may also indicate their integration within local Hungarian diets and institutional food environments accessible to students. This is consistent with previous literature indicating that cereals, particularly refined grains, dominate student diets due to their affordability, versatility, and availability in host countries [30]. The high consumption of vegetables may also reflect their routine inclusion in Hungarian meals and their availability through university dining facilities and retail food outlets.

In contrast, lower consumption rates were observed for fish and other seafood (61.6%) and white tubers and roots (67.9%), indicating variability in the inclusion of certain food groups. Reduced fish consumption may be associated with cost, limited availability, unfamiliar preparation methods, or cultural dietary preferences. Previous studies have shown that international students often modify their intake of familiar foods due to acculturation, accessibility constraints, or perceived cost in the host country [31]. Similarly, lower consumption of white tubers and roots may be associated with adaptation to local dietary patterns that prioritise alternative carbohydrate sources such as bread and pasta [30]. Differences in food availability between students’ home countries and the host-country food environment may further contribute to reduced consumption of culturally familiar staple foods [32].

The high consumption of chicken (92.6%), eggs (92.1%), and rice (90.5%) highlights the participants’ reliance on affordable, accessible, and culturally acceptable staple foods. These items are widely recognised as practical sources of macronutrients and are commonly consumed across diverse populations [33,34]. Additionally, foods such as onions (88.9%), fresh milk (87.9%), and bread (86.8%) were frequently consumed, suggesting a degree of dietary integration combining traditional and host-country food choices [35]. The high intake of these staple and convenience foods may also be associated with simplified meal preparation practices commonly adopted by university students due to time and financial constraints [23].

The frequent consumption of baked goods (80.5%) may suggest a preference for energy-dense and convenience-based food choices. This aligns with previous findings demonstrating increased intake of processed and sugary foods among international students during periods of dietary transition [17,36]. While such foods contribute to overall energy intake, they are often limited in micronutrient content, highlighting a potential area for targeted nutritional interventions [37]. Lower consumption of cottage cheese and kefir may be associated with unfamiliarity with fermented dairy products common in Eastern European diets. Similarly, reduced intake of certain fruits such as guava (31.6%) and watermelon (34.7%) may be influenced by seasonal availability and affordability constraints. This is consistent with previous research identifying access, cost, and seasonal availability as important determinants of fruit consumption among student populations [15,38]. Seasonal variation in fruit availability within Hungary may also have influenced the consumption frequency of certain fruits commonly consumed in students’ countries of origin.

Although the consumption of sugar-sweetened beverages was relatively low (33.7%), energy-dense food choices were still evident. The coexistence of these foods alongside high consumption of staple foods suggests the presence of both favourable and less favourable dietary behaviours within this population. This highlights the complexity of dietary patterns among international students, where food variety may increase alongside the inclusion of less nutritious food items [23]. These findings are consistent with the strong positive correlation observed between FVS and UFCS in the present study and further support the notion that greater food variety does not necessarily correspond to improved dietary quality, particularly within transitional food environments characterised by increased exposure to processed and convenience foods [39].

### 4.3. Socio-Demographic and Behavioural Factors Associated with Food Variety Score

The findings of this study indicate that food variety among international students was associated with a range of sociodemographic and behavioural factors. Monthly income was associated with differences in FVS, with lower-income participants exhibiting reduced food variety compared to higher-income groups. These findings are consistent with previous evidence suggesting that financial resources may be associated with access to a wider range of foods [40]. This may be particularly relevant for nutrient-rich foods such as fruits, vegetables, and animal-source foods. Limited financial resources may also be associated with constraints in purchasing culturally preferred or more diverse food items within the host-country food environment [41]. The positive association between monthly income and FVS remained significant after adjustment for other sociodemographic and behavioural factors, suggesting that financial resources may independently contribute to greater food variety among international students [42].

Employment status was also associated with food variety, with employed participants demonstrating higher FVS compared to unemployed participants. One possible explanation is that employed participants may have had greater financial capacity to purchase a wider variety of foods. However, previous literature suggests that employment may also impose time constraints on food preparation [43]. These constraints may influence dietary behaviours in complex ways. Despite these potential time constraints, employed students in the present study may also have had greater financial flexibility, which could be associated with access to a broader range of food options. The independent association observed in the multivariable model further suggests that employment may contribute to food variety beyond its relationship with income alone [44].

Participants not receiving scholarships exhibited higher food variety compared to scholarship recipients, which may reflect differences in financial flexibility or resource allocation. Similarly, undergraduate students demonstrated higher FVS compared to postgraduate students, which may be associated with differences in lifestyle, time availability, or dietary habits. Previous studies have shown that academic demands and lifestyle factors may influence dietary behaviours among student populations [12]. Postgraduate students may also experience increased academic workload and time pressures, potentially contributing to less varied dietary practices [45].

Participants who had received nutrition education demonstrated higher food variety compared to those without such education. However, this association was not retained in the multivariable model. Interestingly, the sensitivity analysis using the aFVS demonstrated that nutrition education became independently associated with higher food variety after excluding unhealthy food items from the score. This finding suggests that nutrition education may be more closely associated with healthier food choices than with overall food variety. This finding is consistent with literature suggesting that nutritional knowledge may be associated with healthier dietary behaviours, although the observed relationship may be influenced by other sociodemographic and behavioural factors [37].

Participants following a special diet had lower food variety compared to those not following a special diet. One possible explanation is that some dietary practices may be associated with a more limited range of foods consumed, despite potential health motivations. Restrictive dietary practices may also be associated with reduced exposure to a wider range of food items, particularly within unfamiliar food environments where suitable alternatives may be limited or less accessible [46]. The persistence of this association in both the primary and sensitivity analyses suggests that following a special diet may consistently limit the variety of foods consumed, irrespective of whether unhealthy food items are included in the FVS. However, these findings should be interpreted cautiously, as the cross-sectional design of the study does not permit conclusions regarding the direction of the observed associations.

### 4.4. Relationship Between Food Variety and Unhealthy Food Consumption

A very strong positive association was observed between FVS and UFCS (r = 0.930, *p* < 0.001). This finding suggests that greater food variety does not necessarily equate to healthier dietary patterns but may also reflect greater inclusion of energy-dense and nutrient-poor food items [47]. While DD is often associated with improved nutrient adequacy, these results highlight that the inclusion of a wide range of foods may occur alongside increased consumption of less nutritious options.

The strong positive correlation observed between FVS and UFCS should also be interpreted in light of the methodological relationship between the two indicators. Both measures were derived from the same FFQ, and the unhealthy food items included in the UFCS also contributed to the calculation of FVS. Consequently, some degree of overlap between the two measures is expected. Part of the observed correlation may therefore reflect mathematical dependency arising from the shared inclusion of unhealthy food items in the calculation of both indicators.

To evaluate whether this overlap substantially influenced the findings, a sensitivity analysis was performed using the aFVS, which excluded all 30 unhealthy food items included in the UFCS. Despite removing these shared food items, the association between aFVS and UFCS remained strong (r = 0.862, *p* < 0.001). This finding indicates that the observed relationship cannot be explained solely by mathematical overlap between the two measures. Rather, it suggests that participants who consumed a greater variety of healthier foods also tended to consume a greater variety of unhealthy foods.

These findings suggest that greater dietary exposure within this population may include both nutritionally beneficial and unhealthy food choices simultaneously [48]. Among international students, adaptation to a new food environment may therefore be accompanied by broader food exploration that encompasses both healthier foods and highly processed, energy-dense products. The increasing availability and accessibility of convenience foods within university and urban food environments may contribute to this pattern, creating an obesogenic food environment in which greater dietary exposure does not necessarily translate into healthier dietary choices [23].

These findings have important implications for nutrition promotion among international students. Interventions should not focus solely on increasing food variety but should also emphasise the quality of foods selected within a varied diet. Encouraging greater consumption of nutrient-dense foods while reducing the intake of highly processed foods may support healthier dietary adaptation among students living in foreign food environments [45].

### 4.5. Factors Associated with Food Variety Score

The multivariable regression analysis identified several sociodemographic and behavioural factors independently associated with food variety among international students. Higher monthly income and being employed were positively associated with FVS after adjustment for other variables, suggesting that greater financial resources may facilitate access to a wider variety of foods [42,49]. Conversely, scholarship recipients, postgraduate students, and participants following a special diet had significantly lower FVS, indicating that financial circumstances, academic demands, and dietary restrictions may all influence food variety within this population [50].

Although nutrition education was positively associated with FVS, the association did not reach statistical significance in the primary multivariable model. However, the sensitivity analysis using the adjusted Food Variety Score (aFVS), which excluded unhealthy food items included in the UFCS, demonstrated that nutrition education became independently associated with higher food variety. The consistency of the remaining associations across both models further supports the robustness of the observed findings.

Overall, these findings suggest that food variety among international students may be associated with multiple socioeconomic and behavioural factors rather than a single determinant [42,51]. However, because the study employed a cross-sectional design, causal relationships cannot be established, and the observed associations should be interpreted with appropriate caution [52].

### 4.6. Factors Associated with Unhealthy Food Consumption Score

The regression analysis identified several factors associated with unhealthy food consumption. Higher income and employment status were positively associated with unhealthy food consumption, suggesting that increased purchasing power may facilitate access to both healthy and unhealthy food options. Previous studies have similarly reported that higher disposable income may be associated with increased consumption of convenience and processed foods among student populations. Greater financial flexibility may also increase exposure to commercially prepared foods, takeaway meals, and highly processed convenience products commonly available within university and urban food environments [53].

In contrast, scholarship status and level of study were negatively associated with unhealthy food consumption, suggesting that structural and educational factors may influence dietary choices. Participants following a special diet also demonstrated lower unhealthy food consumption, likely reflecting intentional dietary restrictions or health-conscious behaviours. Students adhering to specific dietary practices may be more selective in their food choices and more attentive to food composition, thereby limiting the consumption of highly processed and energy-dense foods [54].

Although nutrition education was positively associated with unhealthy food consumption, the association did not remain statistically significant after adjustment for other sociodemographic and behavioural variables. This finding suggests that nutritional knowledge alone may not be sufficient to influence dietary behaviour in the presence of broader environmental, economic, and lifestyle factors [55].

## 5. Strengths and Limitations of the Study

This study has several important strengths. To our knowledge, it is among the first to examine food variety and unhealthy food consumption concurrently among international students in Hungary. By assessing both overall food variety and unhealthy food consumption, the study provides a more comprehensive understanding of dietary behaviours than studies focusing on a single dietary indicator. An additional strength was the sensitivity analysis using the adjusted Food Variety Score, which demonstrated that the observed associations remained largely consistent after excluding unhealthy food items. This strengthens the robustness of the findings and suggests that the observed relationship between food variety and unhealthy food consumption was not solely attributable to shared food items included in both indicators.

Data collection was conducted over an extended period spanning multiple seasons, which may have influenced food availability and consumption patterns. Seasonal variation was not explicitly accounted for in the analysis, and this may have affected the reported dietary behaviours. Additionally, the use of a cross-sectional study design limits the ability to establish causal relationships between sociodemographic factors and dietary outcomes. The use of purposive and convenience sampling may have introduced selection bias. Consequently, the findings may not be fully representative of all international students studying in Hungary. Therefore, the generalisability of the findings should be interpreted with caution.

The reliance on self-reported dietary data may also introduce recall bias, social desirability bias, and reporting inaccuracies. Furthermore, the use of an FFQ may not fully capture portion sizes or precise quantities of food consumed, potentially affecting the accuracy of dietary intake estimation. The FFQ was adapted from previously validated instruments, reviewed by an expert panel, and pre-tested among international students. However, additional psychometric validation, including test–retest reliability and construct validity, was not conducted within the study population. This should be considered when interpreting the findings.

Another limitation is that both FVS and UFCS were derived from the same FFQ. Although the sensitivity analysis demonstrated that the strong association between these measures persisted after excluding shared unhealthy food items from the aFVS, some degree of methodological dependence cannot be completely excluded. Future studies using independent dietary assessment methods or mutually exclusive dietary indicators may provide further confirmation of these findings.

## 6. Conclusions

The findings of this study indicate that food variety among international students in Hungary varies considerably, reflecting differences in the number of individual food items consumed. Sociodemographic and behavioural factors, particularly employment status, scholarship status, level of study, and dietary practices, were associated with both food variety and unhealthy food consumption, highlighting the complex determinants of dietary behaviour within this population.

Importantly, a very strong positive association was observed between food variety and unhealthy food consumption. The persistence of this association in the sensitivity analysis, which excluded unhealthy food items from the adjusted Food Variety Score, suggests that the relationship was not solely explained by methodological overlap between the two indicators. These findings demonstrate that greater food variety does not necessarily correspond to healthier dietary patterns and highlight the limitations of relying solely on food variety as an indicator of dietary quality, particularly within transitional food environments.

The identification of the most and least frequently consumed food groups and individual food items provides valuable insight into prevailing dietary behaviours among international students in Hungary. Overall, the findings suggest that assessments of dietary quality should consider both the diversity and nutritional quality of foods consumed.

Future research should further explore the relationship between food variety, dietary quality, and food security, as well as the role of lifestyle and environmental factors in shaping dietary behaviours among international students. Longitudinal studies are warranted to clarify temporal relationships and better understand how dietary behaviours evolve over time following relocation to a new food environment.

## Figures and Tables

**Table 1:** Socio-demographic characteristics of international students in Hungary, 2024 (*n* = 380).

Variables	Category	*n*	%
Gender	Male	193	51
	Female	187	49
Age	18–25	207	54
	26–30	129	34
	>30	44	12
Continent of origin	Africa	242	64
	Asia	104	27
	Europe	27	7
	The Americas	7	2
Accommodation	Renting alone	100	26
	Shared housing (free)	20	5
	Shared housing (paid)	169	45
	University dormitory	91	24
Scholarship status	Not on scholarship	170	45
	Scholarship recipient	210	55
Level of study	Postgraduate	180	47
	Undergraduate	200	53
Employment status	Employed	111	29
	Unemployed	269	71
Monthly income (HUF)	≤100,000	77	20
	101,000–200,000	206	54
	201,000–300,000	72	19
	>300,000	25	7

HUF = Hungarian Forint; 1 HUF = 0.0028 USD. Percentages may not total 100% due to rounding.

**Table 2:** Mean Food Variety Score across sociodemographic and behavioural characteristics.

Variable	Category	Mean FVS ± SD	*p*-Value
Monthly income (HUF)	≤100,000	42.5 ± 17.1	<0.001
	101,000–200,000	70.3 ± 27.7	
	201,000–300,000	61.7 ± 23.0	
	>300,000	65.2 ± 19.3	
Employment status	Employed	70.3 ± 27.8	<0.001
	Unemployed	59.5 ± 25.6	
Scholarship status	Scholarship recipient	57.0 ± 24.1	<0.001
	Not on scholarship	69.7 ± 28.0	
Level of study	Undergraduate	66.7 ± 27.7	0.002
	Postgraduate	58.3 ± 24.7	
Nutrition education	Yes	68.1 ± 27.9	0.035
	No	61.0 ± 26.1	
Follows a special diet	Yes	50.0 ± 21.1	<0.001
	No	68.3 ± 27.0	

HUF = Hungarian Forint; 1 HUF = 0.0028 USD. Values are presented as mean ± standard deviation. *p*-values were obtained using *t*-test or ANOVA, as appropriate.

**Table 3:** Correlation between food variety score and unhealthy food consumption score.

Variable	r	*p*-Value
FVS vs. UFCS	0.930	<0.001
aFVS vs. UFCS	0.862	<0.001

r = Pearson correlation coefficient; *p*-values based on two-tailed tests.

**Table 4:** Multivariable linear regression analyses of factors associated with the food variety score.

Variable	β	95% CI	*p*-Value	VIF
**Main Analysis**				
Monthly income (HUF)	3.82	0.69, 6.94	0.017	1.10
Employment status	11.80	6.19, 17.41	<0.001	1.14
Scholarship status	−10.63	−15.57, −5.69	<0.001	1.06
Level of study	−7.99	−13.02, −2.95	0.002	1.11
Nutrition education	5.70	−0.07, 11.47	0.053	1.05
Follows a special diet	−14.92	−20.25, −9.60	<0.001	1.05
Sensitivity analysis (adjusted food variety score)				
Monthly income (HUF)	2.78	0.50, 5.07	0.017	1.10
Employment status	8.77	4.66, 12.88	<0.001	1.14
Scholarship status	−7.50	−11.11, −3.88	<0.001	1.06
Level of study	−5.38	−9.06, −1.69	0.004	1.11
Nutrition education	4.24	0.02, 8.46	0.049	1.05
Follows a special diet	−10.10	−14.00, −6.21	<0.001	1.05

β = unstandardised regression coefficient; CI = confidence interval; VIF = variance inflation factor. Model statistics: FVS model, R^2^ = 0.223, adjusted R^2^ = 0.210; aFVS model, R^2^ = 0.209, adjusted R^2^ = 0.196. Monthly income was entered as a four-category variable and coded as 1 = ≤100,000 HUF, 2 = 101,000–200,000 HUF, 3 = 201,000–300,000 HUF, and 4 = >300,000 HUF. Employment status was coded as 1 = employed and 0 = unemployed. Scholarship status was coded as 1 = scholarship recipient and 2 = not on scholarship. Level of study was coded as 1 = undergraduate and 2 = postgraduate. Nutrition education was coded as 1 = yes and 0 = no. Following a special diet was coded as 1 = yes and 0 = no.

**Table 5:** Multivariable linear regression analysis of factors associated with the unhealthy food consumption score.

Variable	β	95% CI	*p*-Value	VIF
Monthly income (HUF)	1.04	0.08, 1.99	0.034	1.10
Employment status	3.03	1.31, 4.75	<0.001	1.14
Scholarship status	−3.14	−4.65, −1.63	<0.001	1.06
Level of study	−2.61	−4.15, −1.07	<0.001	1.11
Nutrition education	1.46	−0.30, 3.23	0.104	1.05
Follows a special diet	−4.82	−6.45, −3.19	<0.001	1.05

β = unstandardised regression coefficient; CI = confidence interval; VIF = variance inflation factor. Model R^2^ = 0.215; adjusted R^2^ = 0.203. Monthly income was entered as a four-category ordinal variable and coded as 1 = ≤100,000 HUF, 2 = 101,000–200,000 HUF, 3 = 201,000–300,000 HUF, and 4 = >300,000 HUF. Employment status was coded as 1 = employed and 0 = unemployed. Scholarship status was coded as 1 = scholarship recipient and 2 = not on scholarship. Level of study was coded as 1 = undergraduate and 2 = postgraduate. Nutrition education was coded as 1 = yes and 0 = no. Following a special diet was coded as 1 = yes and 0 = no.

## Data Availability

All data generated and analysed during the current study are not publicly available due to the need to protect participants’ privacy and confidentiality, but are available from the corresponding author upon reasonable request.

## Supplementary Materials

The following supporting information can be downloaded at https://www.mdpi.com/article/10.3390/nu18142277/s1. Figure S1. FVS boxplot; Figure S2. Normal Q–Q plot of FVS; Figure S3. Normal Q–Q plot of aFVS; Figure S4. Normal Q–Q plot of UFCS; Figure S5. Histogram of regression standardised residuals for the FVS model; Figure S6. Normal P–P plot of regression standardised residuals for the FVS model; Figure S7. Histogram of regression standardised residuals for the aFVS model; Figure S8. Normal P–P plot of regression standardised residuals for the aFVS model; Figure S9. Histogram of regression standardised residuals for the UFCS model; Figure S10. Normal P–P plot of regression standardised residuals for the UFCS model; Table S1. Food items included in the UFCS; Table S2. Descriptive statistics for FVS, aFVS, and UFCS.

## Abbreviations

The following abbreviations are used in this manuscript:

aFVS	adjusted Food Variety Score
CI	Confidence Interval
DD	Dietary Diversity
FAO	Food and Agriculture Organization
FANTA	Food and Nutrition Technical Assistance III Project
FCS	Food Consumption Score
FFQ	Food Frequency Questionnaire
FVS	Food Variety Score
HUF	Hungarian Forint
MFV	Medium Food Variety
SD	Standard Deviation
SPSS	Statistical Package for the Social Sciences^®^
UFCS	Unhealthy Food Consumption Score
VIF	Variance Inflation Factor
WFP	World Food Programme
