# Supplementary material for: Food Variety and Unhealthy Food Consumption Among International Students in Hungary: Associations with Sociodemographic and Behavioural Factors
Source: Nutrients. 2026 Jul 11;18(14):2277. doi: 10.3390/nu18142277 (PMC13414481; doi:10.3390/nu18142277)
Supplement: Supplementary file 1 [file nutrients-18-02277-s001.zip › nutrients-4375839-supplementary.pdf]

Supplementary Materials

Supplementary Materials: Table S1: Unhealthy food items included in the UFCS; Table S2: Descriptive statistics and normality assessment of the Food Variety Score (FVS), aFVS, and UFCS; Figure S1: Distribution of the FVS; Figures S2–S4: Normal Q–Q plots of the FVS, aFVS, and UFCS; Figures S5–S10: Regression diagnostic plots (histograms of standardized residuals and normal P–P plots) for the multivariable linear regression models using FVS, aFVS, and UFCS as dependent variables.

Supplementary Table S1. Food items included in the Unhealthy Food Consumption Score (UFCS) and used in score construction

**Category**	**Food Item Included in UFCS**
Sweet foods and desserts	Custard
Sweet foods and desserts	Milkshake
Sweet foods and desserts	Ice cream
Sweet foods and desserts	Sweets (gums, candy)
Sweet foods and desserts	Muffins, cupcakes, scones, pastries and tarts
Sweet foods and desserts	Cookies, crunchies and shortbread
Sweet foods and desserts	Energy bars
Sweet foods and desserts	Chocolate
Salty snack foods	Salty snacks (e.g., potato chips, pretzels, corn chips)
Sugar-sweetened beverages	Fizzy drinks and flavoured drinks
Sugar-sweetened beverages	Sugar-sweetened beverages (e.g., lemonade)
High-fat condiments and spreads	Margarine
High-fat condiments and spreads	Coffee creamer
Alcoholic beverages	Wine
Alcoholic beverages	Beer
Alcoholic beverages	Ciders
Alcoholic beverages	Spirits (e.g., vodka, gin, whisky, rum)
Alcoholic beverages	Cocktails
Processed meat products	Processed meat
Processed dairy products	Processed cheese
Fast foods and convenience foods	Deep-fried pastries
Fast foods and convenience foods	Pizza
Fast foods and convenience foods	Pies and sausage rolls
Fast foods and convenience foods	French fries
Fast foods and convenience foods	KFC or similar fried chicken products
Fast foods and convenience foods	Hot dogs
Fast foods and convenience foods	Burgers (non-branded)
Fast foods and convenience foods	McDonald's, Burger King or similar fast-food burgers
Fast foods and convenience foods	Heavy pasta dishes with sauces
Fast foods and convenience foods	Other fast foods ordered or consumed at restaurants
**Total number of food items**	**30**

The Unhealthy Food Consumption Score (UFCS) was derived from 30 food items identified as discretionary foods, processed foods, sugar-sweetened beverages, alcoholic beverages, and fast foods. Consumption of each item during the previous 7 days was coded as Yes = 1 and No = 0. Individual item scores were summed to obtain a total UFCS ranging from 0 to 30, with higher scores indicating greater consumption of unhealthy food items.

Supplementary Table S2. descriptive statistics and normality assessment of the FVS adjusted food variety score, and unhealthy food consumption score among international students in hungary (n = 380)

	**FVS**	**aFVS**	**UFCS**
Mean ± SD	62.69 ± 26.65	45.43 ± 19.33	17.26 ± 8.12
Median	58.00	41.00	17.00
Minimum	13	10	1
Maximum	111	81	30
25th percentile	40.25	29.25	11.00
75th percentile	89.50	65.00	24.00
Shapiro-Wilk p-value	<0.001	<0.001	<0.001

Abbreviations: FVS = Food Variety Score; aFVS = Adjusted Food Variety Score (excluding the 30 food items included in the UFCS); UFCS = Unhealthy Food Consumption Score; SD = standard deviation.

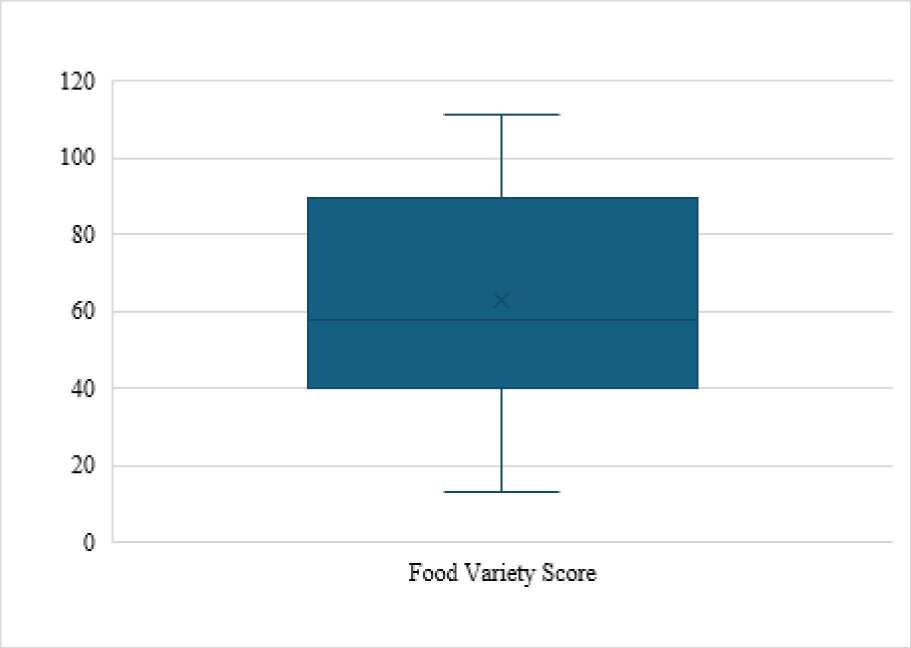

Figure S1. Distribution of FVS shown as a boxplot among international students in Hungary (n = 380)

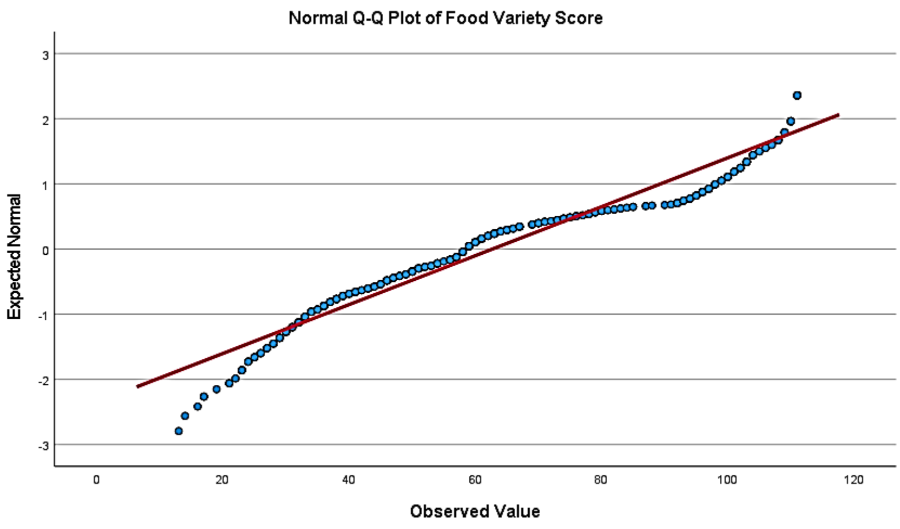

Figure S2. Normal Q–Q plot of the FVS among international students in Hungary (n = 380).

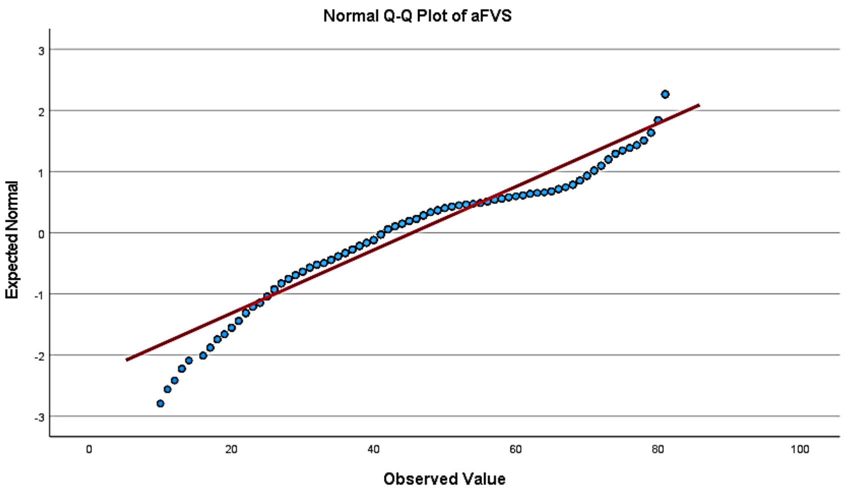

Figure S3. Normal Q–Q plot of the adjusted Food Variety Score (aFVS) among international students in Hungary (n = 380).

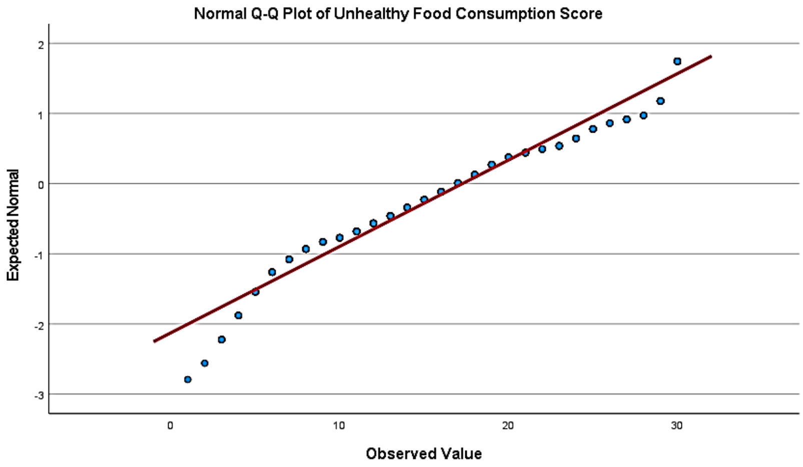

Figure S4. Normal Q–Q plot of the Unhealthy Food Consumption Score (UFCS) among international students in Hungary (n = 380).

Regression diagnostics. The histogram of standardized residuals and normal P–P plot were examined to assess the assumptions of normality for the multivariable linear regression model.

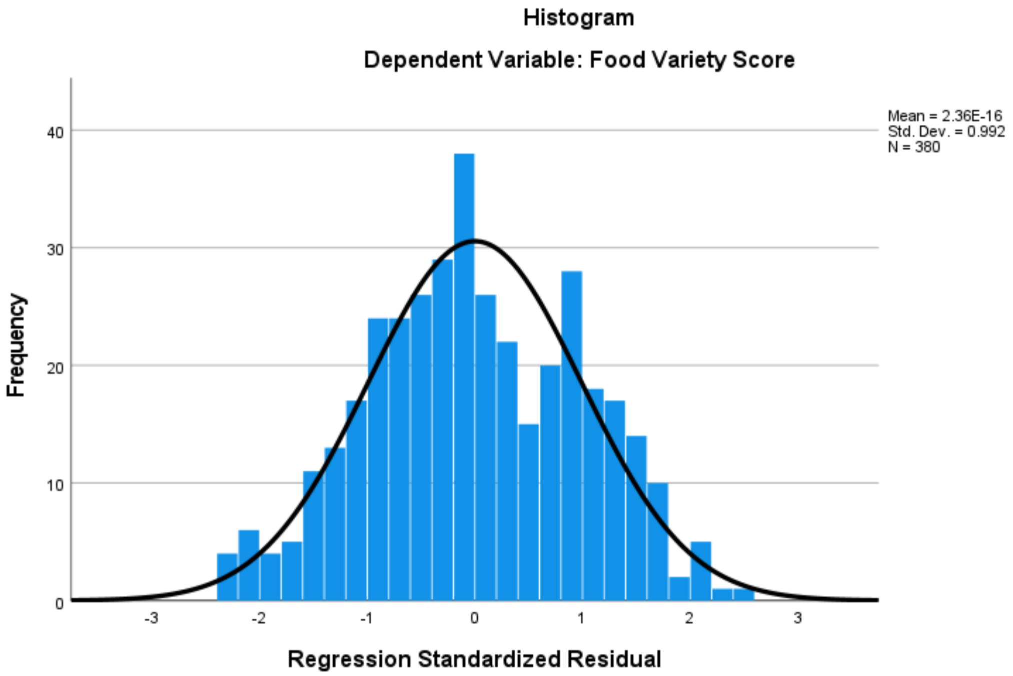

Figure S5. Histogram of standardized residuals from the multivariable linear regression model for the FVS among international students in Hungary (n = 380).

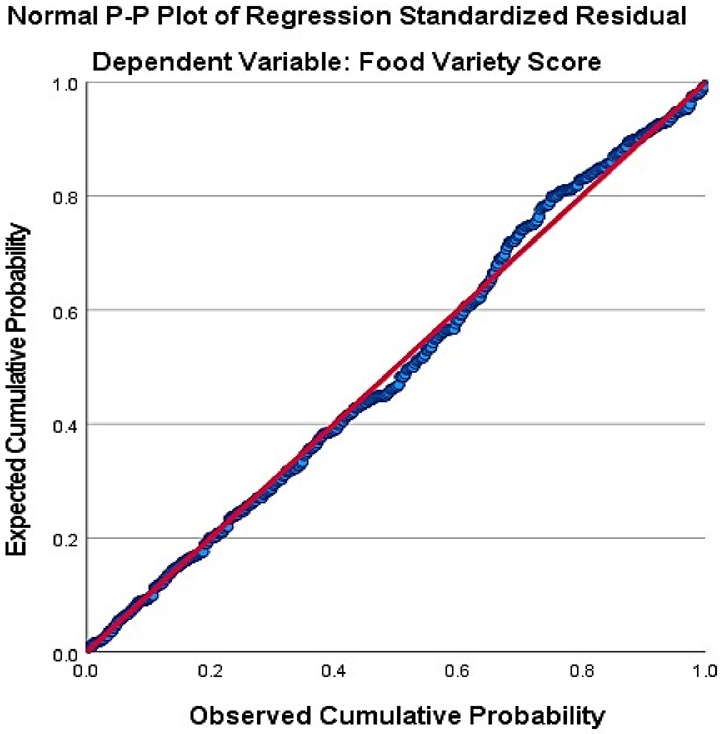

Figure S6. Normal P–P plot of standardized residuals from the multivariable linear regression model for the FVS among international students in Hungary (n = 380)

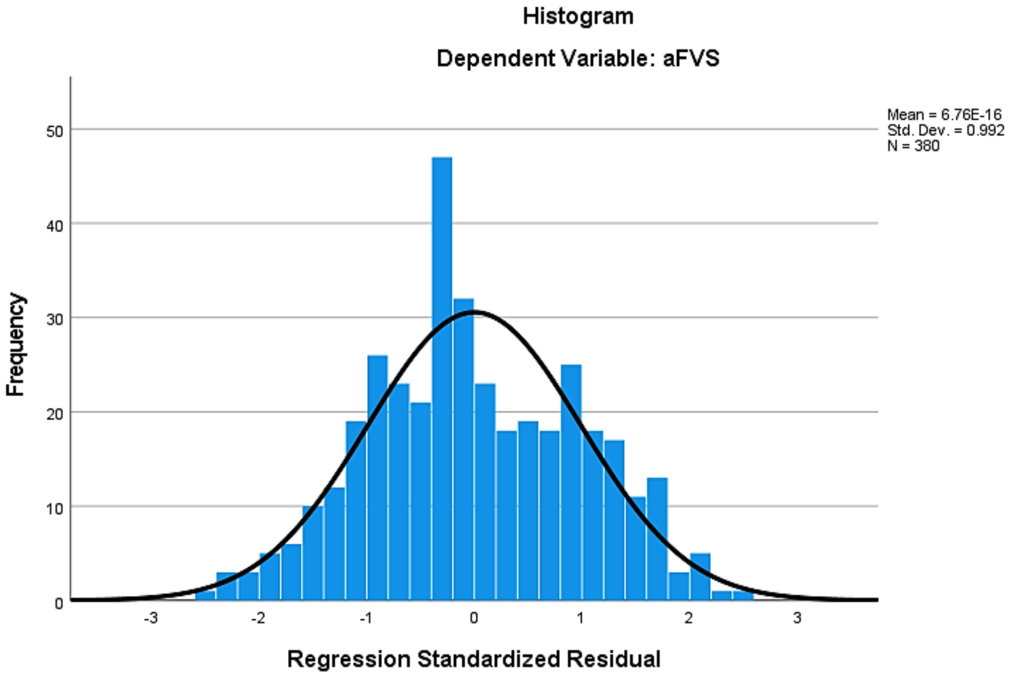

Figure S7. Histogram of standardized residuals from the multivariable linear regression model for the aFVS among international students in Hungary (n = 380).

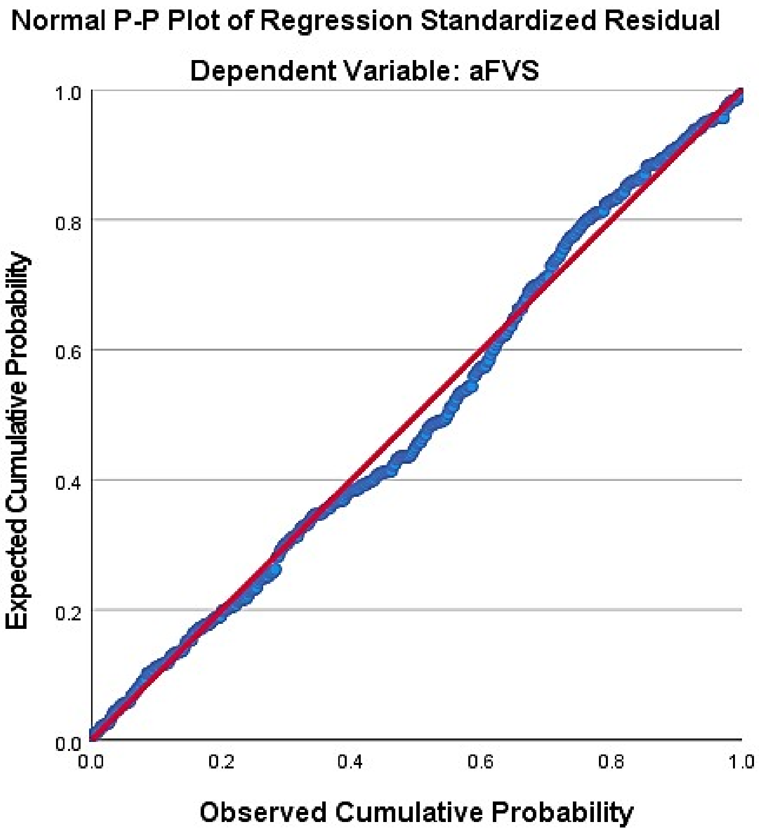

Figure S8. Normal P–P plot of standardized residuals from the multivariable linear regression model for the FVS among international students in Hungary (n = 380)

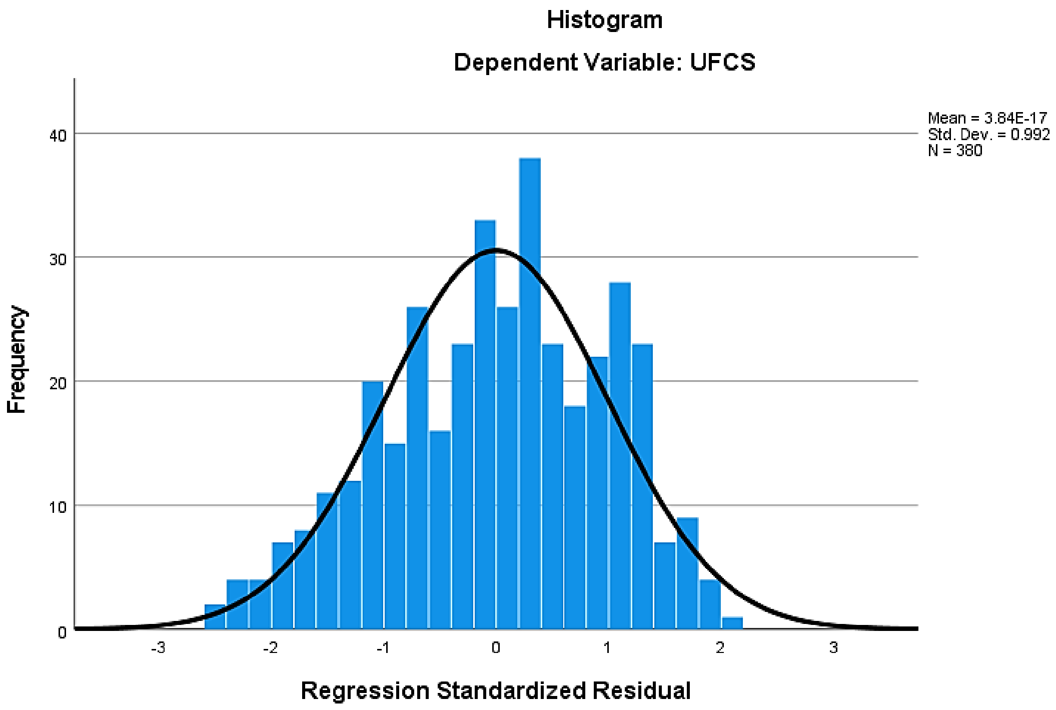

Figure S9. Histogram of standardized residuals from the multivariable linear regression model for the UFCS among international students in Hungary (n = 380).

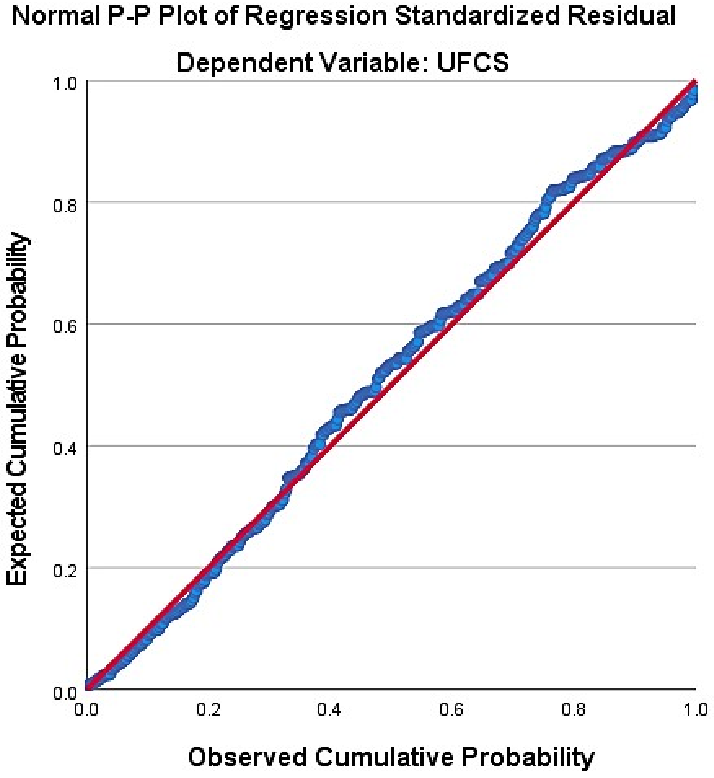

Figure S10. Normal P–P plot of standardized residuals for the multivariable linear regression model with Unhealthy Food Consumption Score (UFCS) as the dependent variable.

Supplementary Questionnaires: Sociodemographic questionnaire and Food Frequency Questionnaire (FFQ) used for data collection.

Food and Nutrition Security and Coping Strategies of International Students in Hungary

Dear Participant,

Thank you for your participation in this study.

My name is Zibuyile Mposula, currently registered for PhD in Nutrition at the University of Debrecen.

By participating in this survey, you will be volunteering to contribute to the research study on the assessment of food consumption habits, food and nutrition security status during as well as the health and lifestyle of international students in Hungary.

Your assistance in the completion of this survey is deeply appreciated. It will take approximately 30 minutes of your time. Your responses will be confidential, and we do not collect identifying information IP address.

Your participation in this research study is voluntary. If you decide to participate in this research survey, you may withdraw at any time. Should you wish to participate in this study, your answers will not be shared with anyone but will be used anonymously for only scholarly purposes by the research team.

* Indicates required question

1. I hereby confirm that I have been informed by the researcher, Zibuyile Mposula, about the nature, conduct, benefits and risks of this study. I have also received, read and understood the Participant Letter of Information regarding the study. I am aware that the results of the study, including personal details regarding my sex, age, date of birth, initials and diagnosis will be anonymously processed into a study report. In view of the requirements of research, I agree that the data collected during this study can be processed in a computerised system by the researcher. I may, at any stage, without prejudice, withdraw my consent and participation in the study. I have had sufficient opportunity to ask questions and (of my own free will) declare myself prepared to participate in the study. I understand that significant new findings developed during the course of this research which may relate to my participation will be made available to me. *

Check all that apply.

- [ ] I agree to participate

SOCIO-DEMOGRAPHIC QUESTIONNAIRE

This questionnaire covers certain aspects of your life, including work and personal details, health and illness, lifestyle and social life that is relevant to health. The answers to these questions will be kept strictly confidential and the personal information such as your name and email address will not be identifiable on any reports or publications.

2. Please enter the USERNAME that has been allocated to you (e.g. FNHU1001). (Please note that your name and personal details will not be mentioned in any of the reports or published. *

____

3. Age Category (in years) *

Mark only one oval.

- ( ) 18-20
- ( ) 21-25
- ( ) 26-30
- ( ) >30

4. Gender *

Mark only one oval.

- ( ) Male
- ( ) Female

5. Race *

Mark only one oval.

- ( ) African/African American
- ( ) Asian (Far East, Southeast Asia, or the Indian subcontinent e.g Cambodia, China, India, Japan, Korea, Malaysia, Pakistan, the Philippine Islands, Thailand, and Vietnam.
- ( ) Native Hawaiian or Other Pacific Islander (A person having origins in any of the original peoples of Hawaii, Guam, Samoa, or other Pacific Islands)
- ( ) White (Original people of Europe, the Middle East, or North Africa)
- ( ) Spanish/Hispanic (A person of Cuban, Mexican, Puerto Rican, South or Central American, or other Spanish culture or origin)

6. Country of origin *

____

7. What is your native language? *

____

8. In which city do you reside in Hungary? *

____

9. What is the name of your university institution? *

10. Please indicate if you are a sponsored (scholarship recipient, non-sponsored student. *

Mark only one oval.

- Scholarship recipient
- Not on scholarship

11. If you are a scholarship recipient, please specify the funding institution from your **home country**. **(Enter 0 if you are not sponsored).** *

12. If you are a scholarship recipient, please specify the funding institution in **Hungary**. **(Enter 0 if you are not sponsored).** *

13. Please specify the level of study you are currently registered for *

Mark only one oval.

- Bachelor
- Masters
- PhD
- Post Doctoral

14. Please describe your living arrangements in Hungary. *

Mark only one oval.

- Living at home
- Renting an apartment by yourself
- Shared apartment
- University dormitory/residence
- Living with a friend or partner (Living for free)

15. Do you have any problems with the state of your accommodation? Please tick what you are having problems with. *

Check all that apply.

- I don't have any problems with my accommodation.
- Structurally related problems (foundation walls, floor joists, rafters, and window and door headers.).
- The place is too small.
- Improper drainage.
- Broken or malfunctioning heating system.
- Poor overall maintenance.
- Plumbing.
- Poor ventilation.

16. How would you describe your current employment status? *

Mark only one oval.

- Student, unemployed
- Internship
- Fixed-term contract / full-time
- Working part-time - (e.g. waiter, movie extra, manual labour, call centre, Wolt, Food Panda. etc.)
- Self-employed (owning a business)

17. If employed, what is your job occupation? (e.g Call Centre Agent; Entrepreneur; Intern; Business Analyst; etc.)

____

18. What is the **estimated** total of your allowance or earnings per month (HUF)? *
This includes stipend, allowance from home, salary, business profit.

Mark only one oval.

- 60000
- 80000
- 100000
- 120000
- 150000
- 200000
- 250000
- 300000
- 350000
- 400000
- 450000
- 500000
- >500000

19. What is the estimated total amount you spend on **accommodation (including utilities)** per month (HUF)? *

Mark only one oval.

- 0
- 40000
- 60000
- 80000
- 100000
- 120000
- 140000
- 160000
- 180000
- 200000
- 220000
- 250000
- 300000
- >300000

20. What is the estimated total amount you spend on food per month (HUF)? *

Mark only one oval.

- 0
- 20000
- 40000
- 60000
- 80000
- 100000
- 120000
- >120000

21. What is the estimated total amount you spend on social events (e.g. movies, parties) per month (HUF)? *

Mark only one oval.

- 0
- 5000
- 10000
- 15000
- 20000
- 25000
- 30000
- 35000
- 40000
- >40000

22. What is the estimated total amount you spend traveling around your city per month (HUF)? *

Mark only one oval.

- 0
- 3000
- 6000
- 9000
- 10000
- >10000

23. What is the estimated total amount you spend on clothes per month (HUF)? *

Mark only one oval.

- 0
- 5000
- 10000
- 15000
- 20000
- > 20000

24. What is the estimated total amount you spend on university extras (e.g. printing, books, stationary) per month (HUF)? *

Mark only one oval.

- 0
- 5000
- 10000
- 15000
- >15000

25. What is the estimated total amount you spend on alcohol per month (HUF)? *

Mark only one oval.

- 0
- 5000
- 10000
- >10000

26. What is the estimated total amount you spend on cigarettes or tobacco per month (HUF)? *

Mark only one oval.

- 0
- 5000
- 10000
- >10000

27. What is the estimated total amount you spend on personal grooming (hair, barber, make-up nails) per month (HUF)? *

Mark only one oval.

- 0
- 5000
- 10000
- > 0000

28. What is the estimated total amount you spend at the gym per month (HUF)? *

Mark only one oval.

- 0
- 5000
- 10000
- 15000
- >15000

29. What is the estimated total amount you send home per month (HUF)? *

Mark only one oval.

- 0
- 10000
- 20000
- 30000
- 40000
- >40000

30. How often do you buy groceries? *

Mark only one oval.

- Once a month
- 2-3 times a month
- 4-5 times a month
- >5 times a month

31. Choose ONE where do you **mostly** buy food? *

Mark only one oval.

- Aldi
- Spar
- Lidl
- Coop
- Tesco
- Auchan
- Penny Market
- Local Shop
- Kiosk
- Other

32. Where do you buy your ethnic food? (Shop name and the city) (e.g. Kashmir Bazar, Budapest) *

____

33. What was the source of the information regarding buying and eating food in Hungary? *

Mark only one oval.

- Word of mouth
- University website
- International office
- I came across it while browsing the city

34. What was the source of the information regarding where you can buy ethnic/traditional food items? *

Mark only one oval.

- Word of mouth
- University website
- International office
- I came across it while browsing the city

35. How do you get to the university? *

Mark only one oval.

- Walk
- Bus
- Tram
- Metro
- Car
- Bicycle
- Taxi/Cab

75. If you smoke or use tobacco, how many times do you do so per day? *

Mark only one oval.

- 0
- 1
- 2-3
- 2-5
- >5

FOOD FREQUENCY QUESTIONNAIRE

Please indicate the food you ate during the past 7 days by ticking the appropriate box, where applicable

76. Meat Group *

Check all that apply.

	Yes	No
**Beef**	☐	☐
**Mutton/Lamb - sheep**	☐	☐
**Pork**	☐	☐
**Goat**	☐	☐
**Dried meat (Traditional Hungarian sausage; Biltong; Jerky etc.)**	☐	☐
**Processed meat (ham, polony, ham, sausages)**	☐	☐
**Ground (minced) meat**	☐	☐
**Chicken**	☐	☐
**Turkey**	☐	☐
**Fresh fish**	☐	☐
**Canned fish (tuna, pilchards/sardines, etc.)**	☐	☐
**Frozen fish**	☐	☐
**Seafood (prawns, mussels, calamari, crab, shrimp)**	☐	☐
**Organ meat (liver, kidneys, hearts, lungs, tripe)**	☐	☐

77. Eggs Group *

Check all that apply.

	Yes	No
**Eggs**	☐	☐

78. Dairy Products *

Check all that apply.

	Yes	No
**Fresh milk**	☐	☐
**UHT (long life milk)**	☐	☐
**Powdered milk**	☐	☐
**Kafir**	☐	☐
**Sour cream (tejfol)**	☐	☐
**Cottage cheese (túró)**	☐	☐
**Other processed cheese**	☐	☐
**Custard**	☐	☐
**Milkshake**	☐	☐
**Ice cream**	☐	☐
**Youghurt**	☐	☐

79. **Cereals and Starch Products** *

Check all that apply.

	Yes	No
**All rice**	☐	☐
**Maize (Maize grits (kukoricadara), Polenta, Samp, porridge, corn on the cob, popcorn, sweet corn)**	☐	☐
**Wheat Grits - Búzadara**	☐	☐
**Pasta/Macaroni/Spaghetti**	☐	☐
**All bread**	☐	☐
**Dumplings/Steamed bread**	☐	☐
**Langos, samosa, doughnut (deep-fried pastries)**	☐	☐
**Breakfast cereals (corn flakes, weet bix, oats, muesli)**	☐	☐
**All tubers (potatoes and similar)**	☐	☐

80. **Lentils and Nuts** *

Check all that apply.

	Yes	No
**All dried beans**	☐	☐
**Canned beans**	☐	☐
**Lentils**	☐	☐
**Nuts**	☐	☐
**Soya**	☐	☐

81. **Vitamin A Rich Food** *

Check all that apply.

	Yes	No
**Green-leafy vegetables**	☐	☐
**Dark-yellow vegetables (carrots, yellow squash, sweet potatoes, pumpkin, butternut)**	☐	☐
**Spinach**	☐	☐
**Apricots**	☐	☐
**Peach (yellow cling)**	☐	☐
**Mango**	☐	☐

82. **Other fruit** *

Check all that apply.

	Yes	No
**Apple**	☐	☐
**Pear**	☐	☐
**Grapes**	☐	☐
**Plum**	☐	☐
**Lemon/lime**	☐	☐
**Orange**	☐	☐
**Tangerine (mandarin)**	☐	☐
**Banana**	☐	☐
**Pineapple**	☐	☐
**Avocado**	☐	☐
**Kiwi fruit**	☐	☐
**Watermelon**	☐	☐
**Guava**	☐	☐
**Other fresh fruit**	☐	☐
**Dried fruit**	☐	☐
**100% fruit juice/ pure fresh fruit juice**	☐	☐

83. **Other Vegetables** *

Check all that apply.

	Yes	No
**Onions**	☐	☐
**Cabbage**	☐	☐
**Beetroot**	☐	☐
**Tomatoes, tomato puree, tomato sauce**	☐	☐
**Green beans (fresh)**	☐	☐
**Peas (fresh)**	☐	☐
**Cauliflower**	☐	☐
**Broccoli**	☐	☐
**Chilli (red/green)**	☐	☐
**Lettuce**	☐	☐
**Green, yellow, red sweet bell pepper**	☐	☐
**Frozen vegetables (mixed)**	☐	☐
**Ginger (fresh)**	☐	☐
**Garlic**	☐	☐

84. **Oils and Fats** *

Check all that apply.

	Yes	No
**Margarine**	☐	☐
**Butter**	☐	☐
**Olive oil**	☐	☐
**Palm oil**	☐	☐
**Other vegetable oil**	☐	☐
**Coconut oil**	☐	☐
**Salad dressing**	☐	☐
**Mayonnaise**	☐	☐
**Coffee Creamer**	☐	☐

85. **Sweets and Other** *

Check all that apply.

	Yes	No
**Sweets (gums, candy)**	☐	☐
**Muffins, cupcakes, scones,pastries, tarts**	☐	☐
**Cookies, crunchies, shortbread**	☐	☐
**Energy bars**	☐	☐
**Chocolate**	☐	☐
**Salty snacks (eg potato chips, pretzels, corn chips)**	☐	☐

86. **Beverages** *

Check all that apply.

	Yes	No
**Fizzy drinks & flavoured drinks**	☐	☐
**Wine**	☐	☐
**Beer**	☐	☐
**Ciders**	☐	☐
**Spirits (vodka, gin, whiskey, rum)**	☐	☐
**Cocktails or other (liqueur)**	☐	☐
**Sugar beverages (e.g lemonade)**	☐	☐
**Coffee**	☐	☐
**Tea**	☐	☐

87. **Fast Food and Similar** *

Check all that apply.

	Yes	No
**Pizza**	☐	☐
**Pies and sausage rolls**	☐	☐
**French fries**	☐	☐
**KFC or similar**	☐	☐
**Grilles chicken, basted with sauce**	☐	☐
**Spicy curry**	☐	☐
**Hot dogs**	☐	☐
**Burger (non-branded**	☐	☐
**Mc Donald's, Burger King**	☐	☐
**Heavy pasta (with sauces)**	☐	☐
**Other fast food (ordered or eaten at a restaurant**	☐	☐
